# Revolution in sepsis: a symptoms-based to a systems-based approach?

**DOI:** 10.1186/s12929-024-01043-4

**Published:** 2024-05-30

**Authors:** Geoffrey P. Dobson, Hayley L. Letson, Jodie L. Morris

**Affiliations:** Heart, Sepsis and Trauma Research Laboratory, College of Medicine and Dentistry, James Cook University, 1 James Cook Drive, Townsville, QLD 4811 Australia

**Keywords:** Sepsis, Intra-abdominal, Infection, Inflammation, Coagulopathy, ALM

## Abstract

Severe infection and sepsis are medical emergencies. High morbidity and mortality are linked to CNS dysfunction, excessive inflammation, immune compromise, coagulopathy and multiple organ dysfunction. Males appear to have a higher risk of mortality than females. Currently, there are few or no effective drug therapies to protect the brain, maintain the blood brain barrier, resolve excessive inflammation and reduce secondary injury in other vital organs. We propose a major reason for lack of progress is a consequence of the treat-as-you-go, single-nodal target approach, rather than a more integrated, systems-based approach. A new revolution is required to better understand how the body responds to an infection, identify new markers to detect its progression and discover new system-acting drugs to treat it. In this review, we present a brief history of sepsis followed by its pathophysiology from a systems’ perspective and future opportunities. We argue that targeting the body’s early immune-driven CNS-response may improve patient outcomes. If the barrage of PAMPs and DAMPs can be reduced early, we propose the multiple CNS-organ circuits (or axes) will be preserved and secondary injury will be reduced. We have been developing a systems-based, small-volume, fluid therapy comprising adenosine, lidocaine and magnesium (ALM) to treat sepsis and endotoxemia. Our early studies indicate that ALM therapy shifts the CNS from sympathetic to parasympathetic dominance, maintains cardiovascular-endothelial glycocalyx coupling, reduces inflammation, corrects coagulopathy, and maintains tissue O_2_ supply. Future research will investigate the potential translation to humans.

## Introduction: a global perspective

At the present time there is no magic bullet or pharmacological therapy for controlling the bioburden of propagating inflammation from intra-abdominal sepsis.

Coccolini and colleagues (2023) [26]

Sepsis is recognised by the World Health Organization (WHO) as a global health priority across all countries and ages [26]. It is the most common cause of admission and death in the Intensive Care Unit (ICU) [91]. Each year, ~ 49 million are afflicted and 11 million patients die, with the majority occurring in low- and middle-income countries [142]. Nearly half, ~ 20 million cases, occur in children under 5 years of age, with ~ 2.9 million deaths [91, 142]. *These global mortality numbers translate to* ~ *1200 deaths per hour or one death every 3 s.* The most common causes are infections of the respiratory tract (up to 50%), followed by the abdomen, bloodstream, renal system, skin and central nervous system (CNS) [177]. Males appear to have a higher risk of mortality than females [122], which may be due to females having a more robust cell-mediated immune response [144]. Sepsis continues to pose a significant threat to the senior population with their lower physiological reserves and multiple comorbidities [80].

Sepsis is a clinical syndrome that develops from a dysregulated host response to infection [68, 91]. It is characterized by a systemic inflammatory response syndrome (SIRS) comprising hyperinflammation, immunosuppression, immune paralysis and multiple organ dysfunction syndrome (MODS) [91, 177]. Septic shock is a further complication that leads to persistent hypotension, widespread tissue hypoperfusion, SIRS, MODS and an altered mental state [4, 22, 56, 91]. Modern core definitions of sepsis emphasize a more systemic pathobiology with underlying sub-phenotypes, each potentially requiring different management strategies [148]. These new sub-phenotypes were recently identified by Seymour and colleagues, who used artificial intelligence and machine learning clustering techniques of multiple data sets from over 20,000 patients [148]. The different phenotypes appear to reflect different patient responses to infection that may be associated with varying degrees of CNS hyperactivation, inflammation, immune dysregulation, cardiac depression, endothelial-glycocalyx activation, coagulopathy and organ supply–demand imbalances*.* Today, diagnosing and treating sepsis begins with identifying the type of infection, measuring the host response using biomarkers, such as C-reactive protein, procalcitonin and lactate, predicting the likelihood of organ dysfunction, fluid therapy and possible drainage/surgery for source control [91, 142]. After providing a brief history of sepsis, we will discuss its pathophysiology from a systems' perspective, and the challenges and opportunities for the twenty-first century.

## Brief history

The medical profession will make early diagnosis, will insist on early intervention, will limit its surgical procedures to the least possible handling and trauma consistent with closure of the opening and relief of pus tension, will limit the duration of anaesthesia and the amount of the anaesthetic, will shorten the actual time of operation, will insure the continued absence of pus tension, will eliminate the sepsis already in the blood, restore the blood pressure and will inhibit absorption by position*.*

John Murphy (1908) [120] p872

**Fig. 1:**
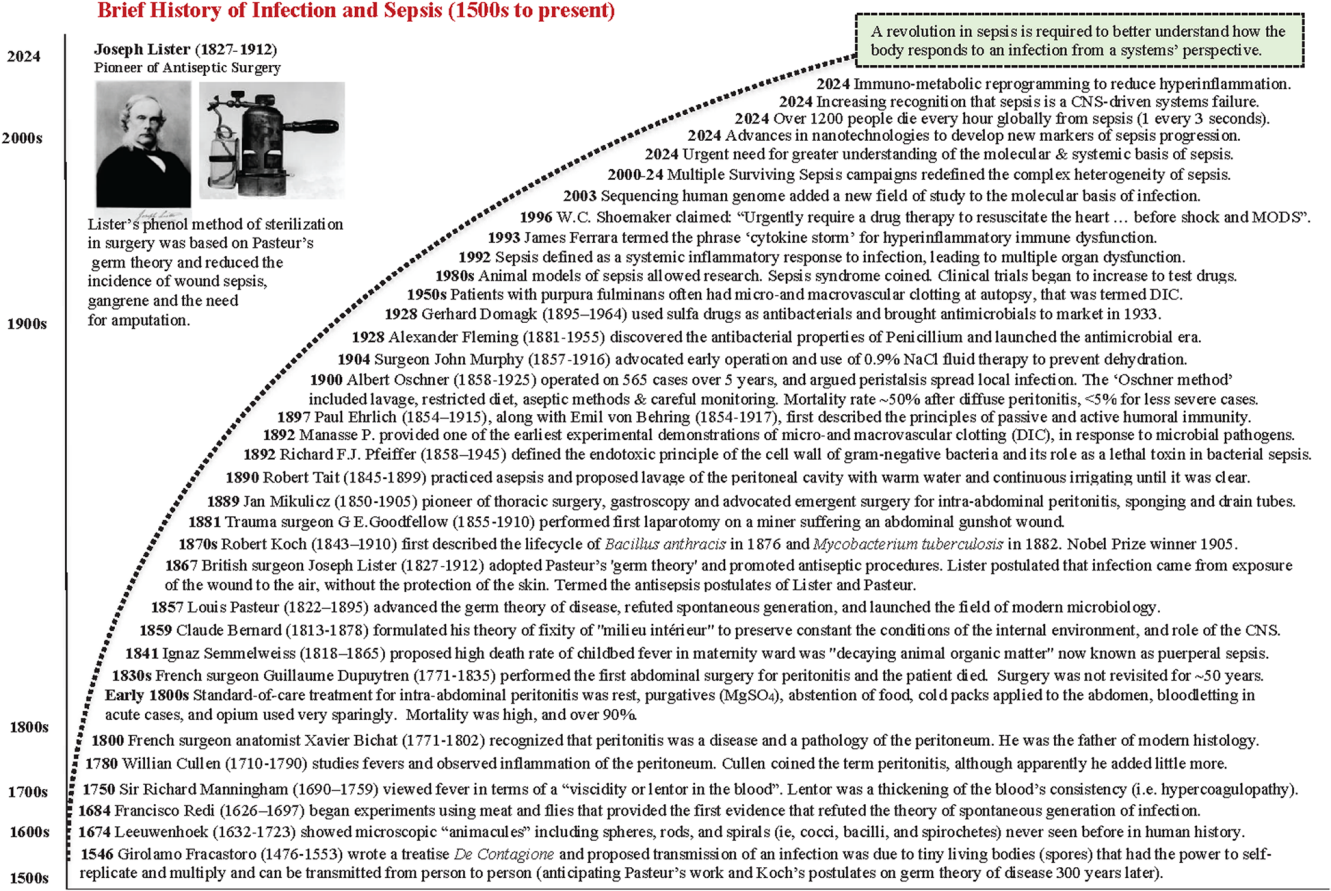
Brief history of infection and sepsis from the Renaissance to the present. Note the advances in the 19th century when asepsis, surgical practice and research were rapidly being developed for major diseases and trauma. The timeline provides a perspective of the changing ideas, practices, and outcomes from which the current thinking and treatments have developed (See text). CNS, central nervous system; MgSO_4_, magnesium sulfate

Pioneer surgeon John Murphy (1857–1916) wrote this description on how to treat a patient with perforative peritonitis over 100 years ago. When we study the history of medicine, one is humbled by how far we have come in advancing knowledge, on one hand, and appreciate the long road ahead to improve current practices, on the other (Fig. 1). Despite flares of brilliance from ancient times to the renaissance, major strides did not occur until the mid-1800s when knowledge and practice became more evidence-based [40, 60, 158]. Louis Pasteur’s and Robert Koch’s germ theory of diseases, Rudolf Virchow’s medicine and cellular pathology and Claude Bernard’s unifying concept of the *internal milieu* all formed the basis of the modern era [60] (Fig. 1). During the latter half of the 19th century, emergent surgery for intra-abdominal peritonitis with drain tubes was advocated by Johann von Mikulicz; Robert Tait practiced aseptic techniques and lavage of the peritoneal cavity; Joseph Lister made great strides in perioperative infection-control, and John Murphy incorporated most in his surgical practice (quote above), including the use of 0.9% saline infusions to avoid dehydration (Fig. 1). Despite these advances, sepsis mortality remained high (> 70%) [60, 127, 158].

This clinical landscape changed in 1928, when Alexander Fleming discovered Penicillium, which launched the modern antimicrobial era [127] (Fig. 1). Advances in bacteriology and antibiotic use in the 1940s and 1950s resulted in a slight mortality reduction from sepsis and septic shock [60, 158]. In the 1960s, the molecular revolution led to an increased understanding of the underlying pathology of infection and clinical trials targeted blunting the immune and inflammatory responses [60]. In the 1980s, animal models of sepsis were introduced into basic research. Today, despite new molecular-based technologies, nanotechnology diagnostics, thousands of research papers and hundreds of clinical trials, unacceptably high mortality rates still remain [27, 155]. *A new revolution is required to better understand how the body responds to an infection, identify new markers to detect its progression and discover new system-acting drugs to treat it.*

## Pathophysiology from a system’s perspective

We begin our systems analysis of sepsis with the central nervous system (CNS) as it is the hierarchical controller of whole-body homeostasis through the multiple feedback circuits (or axes) linking the O_2_ we breathe to mitochondrial ATP production. The term ‘systems’ refers to the whole body’s response to an infection, which includes its activation, progression and outcome [51–53]. A systems-acting drug is defined as one that treats the pathophysiological response from a systems’ perspective (see later).

### CNS sympathetic hyperactivity: major controller of pathophysiology

Sepsis-associated encephalopathy is a diffuse brain dysfunction that occurs secondary to infection in the body without overt CNS infection.

Gofton and Young (2011) [62]

**Fig. 2:**
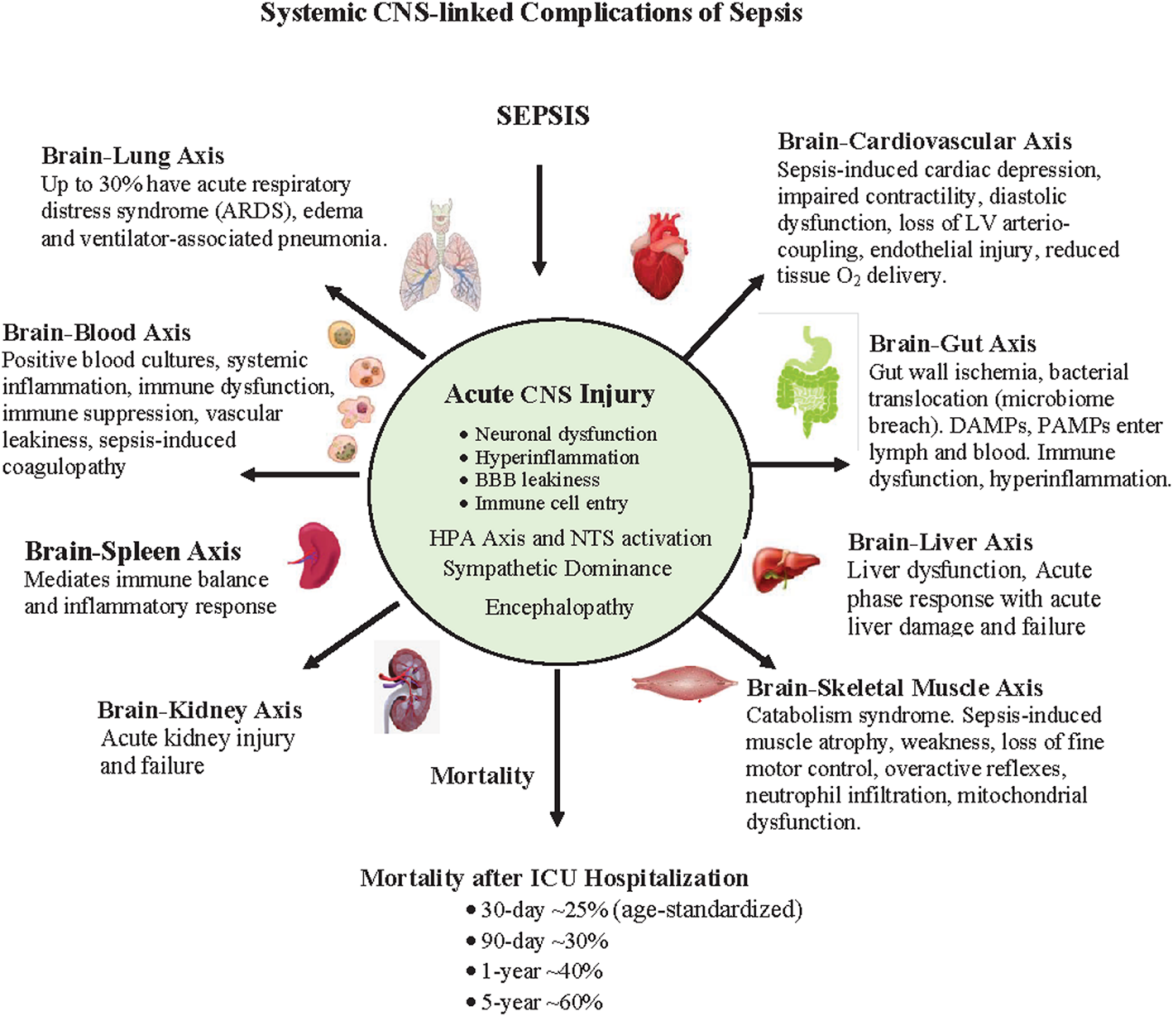
Schematic of CNS-control linked complications following a major infection and sepsis. Excessive inflammation and tissue damage can lead to CNS dysfunction, pulmonary injury, cardiovascular uncoupling, endothelial activation, tissue ischemia, microbiome composition changes, mitochondrial dysfunction, multiple organ failure, and ultimately death. Mortality rates are from Skei and colleagues [155]. Hyperinflammation, immune dysfunction, endotheliopathy, coagulopathy and multiple organ dysfunction are all under the control of the CNS. CNS, central nervous system; BBB, blood–brain barrier; NTS, nucleus tractus solitarius; HPA, hypothalamic-pituitary axis; LV, left ventricle; DAMPs, damage-associated molecular patterns; PAMPs, pathogen-associated molecular patterns

During an infectious challenge, immune cells and their inflammatory products modulate the hypothalamic–pituitary–adrenal (HPA) axis and activate sympathetic stress response via the nucleus tractus solitarius (NTS) [42, 49, 54, 153]. In sepsis, the CNS balance switches to sympathetic dominance with suppression of parasympathetic outflows [7, 96]. This promotes a systemic pro-inflammatory state because the parasympathetic system normally keeps inflammation at bay via activation of vagal cholinergic neurons and splanchnic/splenic nerves, known as the inflammatory reflex [78, 132, 170]). This shift in CNS balance also impacts on multiple brain-axes including the lung, heart and vasculature, gut microbiome, liver, spleen, kidney, lung and muscle (Fig. 2). Animal studies, for example, have shown that blockade of the brain renin–angiotensin–aldosterone system appears to prevent sympathetic hyperactivity and markedly attenuates LV dysfunction during sepsis [36]. Targeting the CNS to reduce its sympathetic discharge offers a potential target for future system-based therapies [96].

In addition to increased sympathetic discharge, sepsis affects brain function through neural afferents, hormones and signals from systemic immune cells and tissues (see below), which can enter the brain via a leaky blood–brain barrier (BBB) (Fig. 2) [124, 146]. This creates a hostile CNS environment of inflammation, oxidative stress and redox imbalance, which can activate glial cells, constrict the microcirculation and cause ischemia, hypoxia and structural nerve damage [104, 183]. In severe cases, the bombardment of injury signals can cause a condition known as sepsis-associated encephalopathy (SAE), defined as diffuse brain dysfunction secondary to sepsis with manifestations ranging from delirium to coma [62, 146, 183]. SAE occurs in up to 70% of septic shock patients, especially in the elderly, neonates, and patients with chronic illness [62]. Furthermore, SAE is a common cause of long-term neurological damage, such as anxiety, memory impairment, and consciousness disorders following severe sepsis [62]. Potential therapeutics could target the maintenance of BBB integrity to reduce the entry of immune cells and their products, and possibly reduce neuroinflammation and the incidence of SAE.

**Fig. 3:**
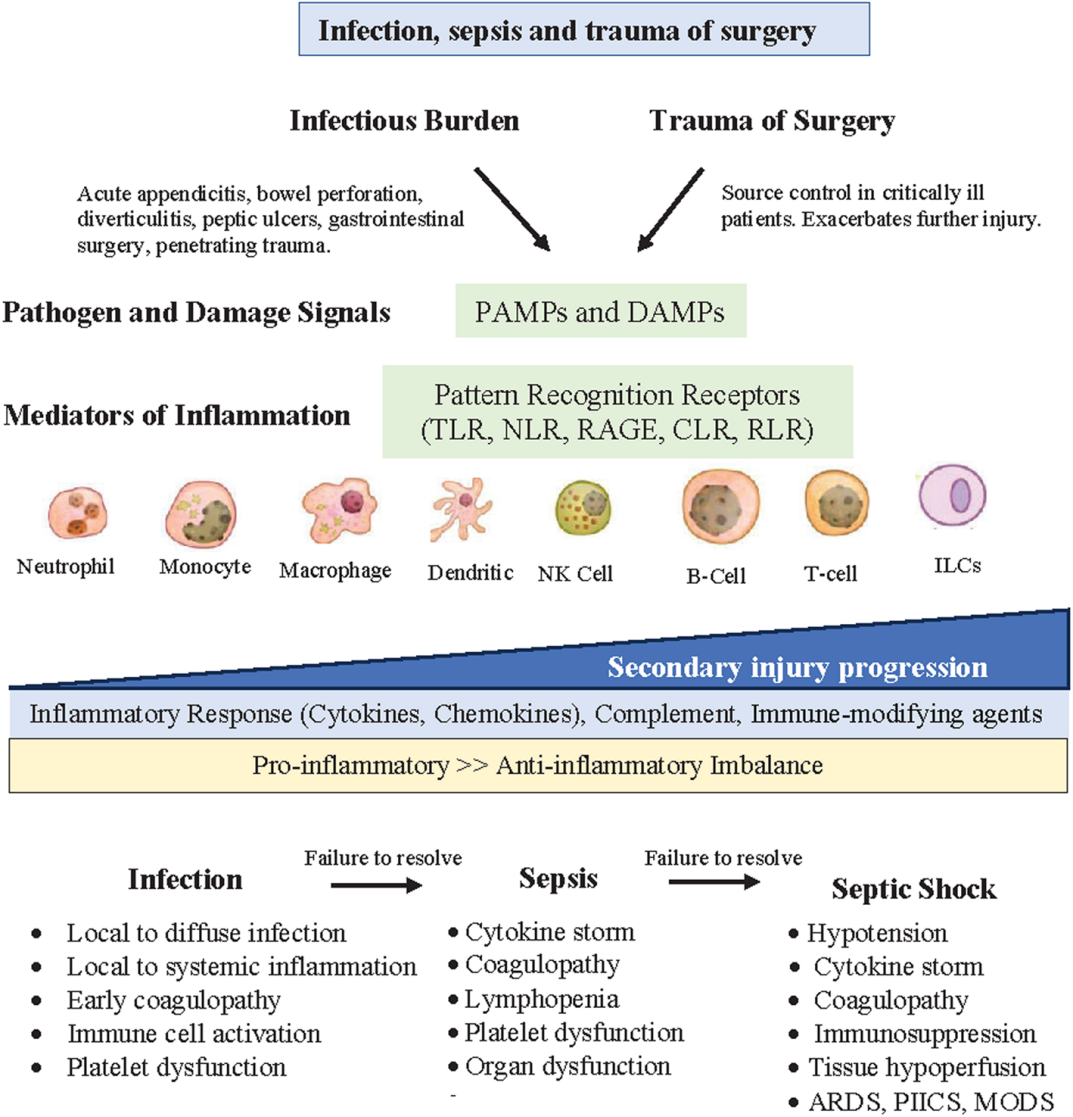
A schematic of the source of pathogen-associated molecular patterns (PAMPs) and damage-associated molecular patterns (DAMPs) in response to an infection, sepsis or the trauma of surgery. The immune-driven inflammatory response is determined by the mix of cytokines and other neural and inflammatory mediators that determine the selection, activation, recruitment and fate of immune effector cells. Secondary injury is defined as a *progressive* process that begins with a pathogen or injury and leads to CNS dysfunction, excessive inflammation, immune dysfunction, coagulopathy, oxidative stress and mitochondrial energy deficit. Sepsis progresses in the setting of hyperinflammation, immune dysfunction, oxidative stress and redox imbalance. TLR, toll-like receptor; NLR, NOD-like receptor; RAGE, receptor for advanced glycation end products; CLR, C-type lectin-like receptor; RLR, RIG-I-like receptor; NK cell, natural killer cell; ILC, innate lymphoid cell; ARDS, acute respiratory distress syndrome; PIICS, Persistent Inflammation, Immunosuppression, and Catabolism Syndrome; MODS, multiple organ dysfunction syndrome

Increased CNS-sympathetic outflows may also reduce mesenteric blood flow to the gut [37, 177]. This is particularly relevant in sepsis because if the gut becomes ischemic, it can become leaky and bacteria or their active metabolic products (lipopolysaccharides, cytokines, neuropeptides, and protein messengers) can enter the mesenteric lymph or bloodstream and exacerbate the infectious load, increase immune dysfunction, heighten inflammation, worsen coagulopathy, and trigger immunosuppression and MODS [25, 37, 75, 156, 177]. The sepsis-induced derangements in microbial balance can themselves have a profound influence on immune function and cause harm to the host (Fig. 3) [89, 157]. Change in the host’s gut microbiome is bidirectionally linked to the CNS through vagal afferents, immune, and HPA axis modulation, and the CNS in turn can modulate the gut and enteric nervous system [32, 45, 101, 113, 117, 166]. More studies are urgently required to examine the gut-brain axis and microbiome compositional changes during sepsis, which may offer potential targets for future therapeutics [25].

### Host response to infection: immune cell activation and inflammation

Except on few occasions, the patient appears to die from the body’s response to infection rather than from it.

Sir William Osler (1904) [128]

William Osler’s statement over 100 years ago remains the cornerstone of our thinking today. It is the body’s *response to infection* that is the main determinant of outcome, not the bacterial challenge per se. This helps to explain why a sepsis patient, despite successful removal of infectious foci, often fails to respond to ongoing treatments [27]. The early systemic response to an infection involves recruitment of inflammatory leukocytes to phagocytose the invading bacteria and associated cellular debris. Initially, neutrophils, which reside in large numbers in the circulation, are recruited and quickly followed by bone marrow-derived monocytes and macrophages to resolve the threat and restore homeostasis [42, 139]. Other immune cells are also recruited, such as dendritic cells, natural killer (NK) cells, B-cells, T-cells and innate lymphoid cells (ILC) [6, 22], however, their involvement in sepsis is beyond the scope of the present review (Fig. 2).

If an infection, like sepsis, overwhelms the body’s defences, innate immune cells continue to infiltrate the vital organs of the body, including the brain, resulting in collateral tissue damage from cytokines, immune modulators, complement, oxidants, proteases, and toxic extracellular traps (Fig. 2) [84]. The cytokine ‘storm’ that ensues comprises a profound excess of proinflammatory over anti-inflammatory cytokines release into the circulation (Fig. 2). Key mediators include interleukin (IL-1β), tumor necrosis factor (TNF)-α, IL-6, IL-4, IL-8, IL-17, IL-18, and IL-10, [152], as well as platelet-activating factor, complement factors and reactants of coagulopathy and fibrinolysis [179]*.* If concomitant anti-inflammatory processes are not activated sufficiently, the cytokine storm is often followed by persistent lymphopenia and immunosuppression or paralysis, which involves metabolic reprogramming of many different cell types, immune cell depletion, cellular apoptosis and T cell exhaustion [49, 54, 110, 177]. A number of potential targets for resolution of inflammation are underway [138, 186]). Razazi and colleagues, for example, have identified in septic shock patients that activation of the IL-17/interferon (IFN) pathway and vascular endothelial growth factor (VEGF) strongly correlates with i) early sepsis resolution (reduced lactate) and ii) improved ICU survival [138]. Another target area that appears to show promise is metabolic reprogramming of immune cell populations for switching the host’s injury phenotype to a healing one [92, 102, 103].

### Source of pathogen- and injury-generated signals

The two main sources of molecular signals in sepsis that activate early immune cell recruitment and drive inflammation are: 1) pathogen-associated molecular patterns (PAMPs) from the invading infectious microbes, and 2) damage-associated molecular patterns (DAMPs) from tissue injury [42, 177] (Fig. 3). PAMPs and DAMPs are evolutionary conserved motifs that make foreign pathogens or tissue damage recognizable by the host [42, 82, 112]. PAMPs include bacterial lipopolysaccharide (LPS), flagellin and lipoteichoic acid, viral RNA and DNA, surface glycoproteins, lipoproteins, and other membrane components [10, 82, 177]. DAMPs from damaged cells and tissues caused by the infection include fibrinogen, annexins, platelet components, fibronectin, S100 proteins, syndecan-1, F-actin, adenosine triphosphate (ATP), histones, deoxyribonucleic acid (DNA), mitochondrial transcription factor A (TFAM), mitochondrial reactive oxgen species (mitoROS), cytochrome C, IL-1α, high mobility group box protein 1 (HMGB1), heparan sulfate, tenascin C, defensins, amyloid-*β,* and many others [141]. Another source of PAMPs and DAMPs are derived from extracellular vesicles (EVs) released by pathogens, immune or endothelial cells, platelets and damaged host cells [15, 165, 174].

How do host immune cells sense danger? PAMPs and DAMPs are detected by the host’s pattern recognition receptors (PRRs) located on the surface or in the cytoplasm of immune cells [160, 177]. PRRs are the body’s “sensors” of a threat and communicate it to the host via immune cell activation. These sensors include toll-like receptors (TLRs), RIG-I-like receptors (RLRs), NOD-like receptors (NLRs), C-type lectin-like receptors (CLRs), receptors for advanced glycation end products (RAGE) and cytosolic DNA sensors (Fig. 3) [1, 86, 173]. Another important PRR that senses a wide range of PAMPs and DAMPs is the NLR family pyrin domain containing 3 (NLRP3) protein that activates a cytoplasmic multiprotein platform assembly known as the inflammasome [85], which if overexpressed can lead to the death of the patient [181]. *Importantly, PAMPs and DAMPs are not mutually exclusive, and immune cells may express co-receptors and accessory molecules that form ‘partnerships’ to coordinate an immune response* [134]. The extra barrage of DAMPs from an emergent laparotomy, if required, in a sepsis patient is an additional burden rarely mentioned as a potential target to improve outcomes [168] (see later). Identifying the different PAMPs and DAMPs in the blood of sepsis patients may offer a new early diagnostic treatment window for personalized care *before* the cytokine storm develops or tissue blood flow and O_2_ becomes limiting [15, 165, 174].

### Endothelial-glycocalyx: a sensor and effector of immune activation

In acute inflammation, we find, as a general rule, vascular dilatation accompanied by an active condition of the endothelium of the vessel-walls and an exudation with diapedesis, that is to say, three events which concur in producing a considerable afflux of leucocytes towards the injured spot.

E. Metchnikoff (1893) [116] p171

During sepsis, immune cells and their inflammatory products activate the endothelium that result in shedding of its ‘fuzz-like’ glycocalyx, indicated by serum elevations in syndecan-1 and soluble-thrombomodulin [81, 83, 179]. This is called sepsis-induced endotheliopathy [83]. The glycocalyx is negatively charged and anchored to the single layer of endothelial cells that forms the nexus between the vasculature and the tissues [88, 176]. It covers a vast surface area of over 55,000 m^2^ [49, 54]. *During sepsis, the activated endothelium becomes more adhesive, leaky, pro-apoptotic, pro-inflammatory, pro-thrombotic and vasoactive* (Fig. 3) [81, 83, 105, 123]. Glycocalyx shedding is facilitated by stress-activated membrane-bound enzymes, called sheddases, in response to pro-inflammatory cytokines (e.g. TNF-α and IL1-β), reactive oxygen species (ROS) (e.g. superoxide, hydroxyl radical), and by aggressive fluid resuscitation [171]. Of clinical significance, it appears that glycocalyx shedding can repair itself quickly [185]. Luft states that “these cells usually are able to replace their missing coats in a matter of minutes” [106]. However, very little is known about the loss and recovery of the glycocalyx during an infection or sepsis [171].

In critically ill patients, sepsis-induced endotheliopathy is sometimes associated with vascular microthrombosis mediated by platelet activation and the endothelial release of von Willebrand factor (vWF) multimers, which in turn impairs O_2_ delivery to mitochondria. Excessive production of vWF multimers become anchored to endothelial cells as elongated strings and form platelet-vWF complexes known as “microthrombi” [23, 81]. If the pathology becomes more diffuse and systemic, it can lead to a lethal condition known as disseminated intravascular coagulopathy (DIC) (see below). Reducing the early activation of the endothelial glycocalyx or facilitating rapid recovery after shedding may be potential targets for new therapeutics.

### Sepsis-induced coagulopathy: a dynamic entity that evolves over time

Reconstituted systems are as realistic as our insight into the clotting mechanism allows: extrapolation to physiology should therefore be regarded with due suspicion.

Hemker and colleagues [76], p171

**Fig. 4:**
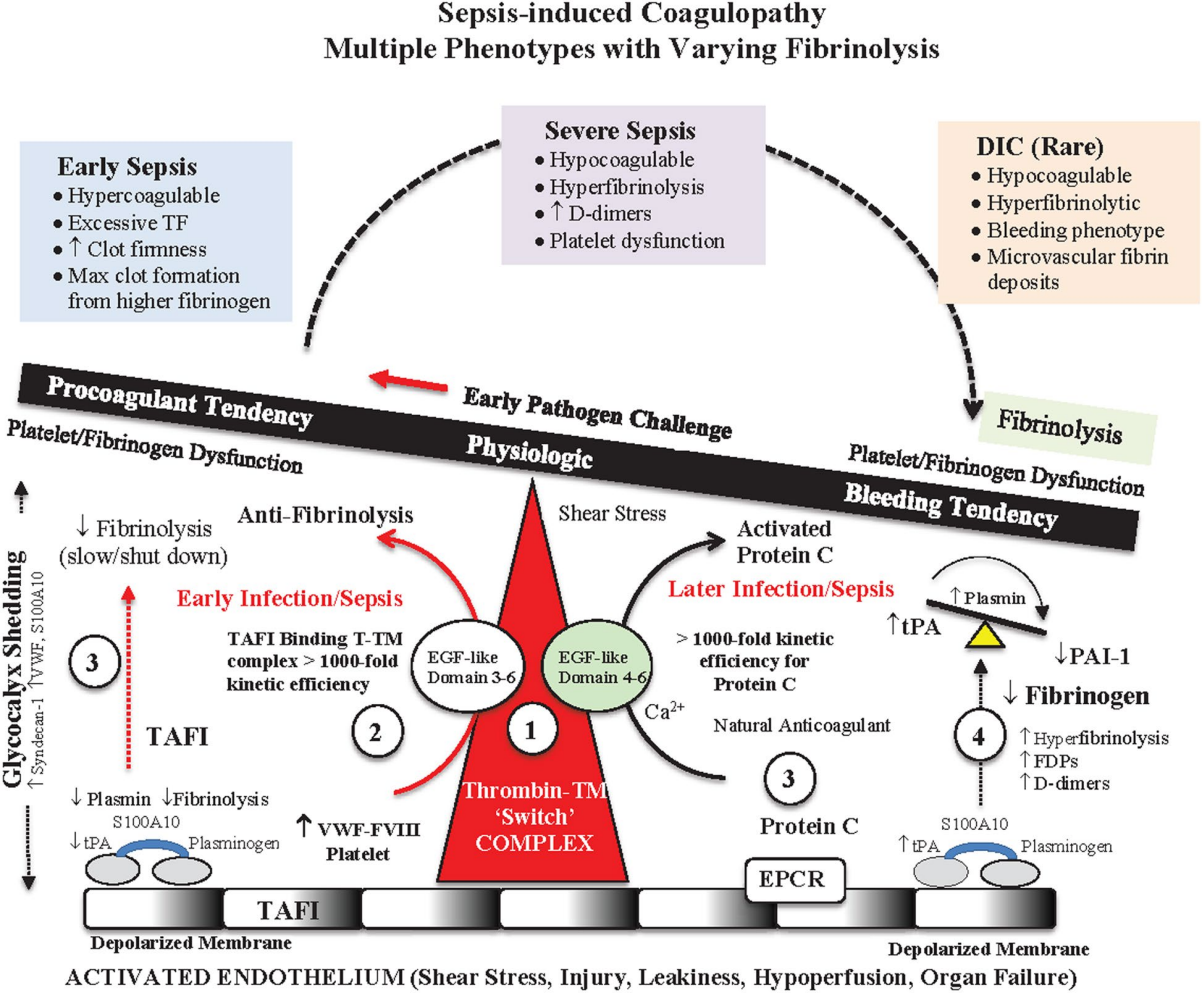
Coagulopathy is a systemic pathological condition in which the blood’s ability to clot is impaired with varying degrees of fibrinolysis. The schematic illustrates the different sepsis-induced phenotypes around the Thrombomodulin (TM)-thrombin switch (1) [47, 48]. The TM-thrombin “switch” regulates coagulation and fibrinolysis in both directions depending on different activators and inhibitors at the thrombin-TM active sites (EFF-like domains) [47, 48]. During an early infection, patients appear to have a procoagulable phenotype which may form from activation of Thrombin-Activatable Fibrinolysis Inhibitor (TAFI) (2), which decreases plasmin levels (3) and increases fibrinogen to form a stronger a stronger clot. As infection progresses the phenotype may change to a more hypocoagulable state where fibrinogen is decreased, D-dimers increase (fibrinolysis), and in extreme cases progresses to a specific hypocoagulation dominated by hyperfibrinolysis with microvascular fibrin deposits (DIC). The phenotypic change from a hyper- to hypo-coagulable state to disseminated intravascular coagulopathy (DIC) appears to be associated with a transition from a TF-dominated inflammatory microenvironment, favoring EGF-like Domain 3–6), to a non-TF dominated environment, favoring EGF-like Domain 4–6, with high mortality. This hypothesis requires knowledge of cytokines, immune cells, tPA, PAI-1, α_2_-antiplasmin, fibrinogen, TAFI levels and remains to be tested. Drugs to modulate the thrombin-TM “switch” following infection and sepsis are urgently required. TPA: tissue plasminogen activator; PAI-1: plasminogen activator inhibitor-1; WVF: Von Willebrand factor; S100A10: S100 calcium binding protein A10; FVIII: Factor VIII; EPCR: endothelial protein C receptor; FDP: FDP: fibrin degradation product

CNS dysfunction, inflammation, endotheliopathy and coagulopathy are all functionally linked through common pathways involved in the regulation of tissue factor (TF) [23, 47, 48, 100, 178, 179]. The common pathways include the TF inhibition pathway, platelet inhibition pathway, the heparin-antithrombin III system, thrombomodulin/protein C system and fibrinolytic pathways (Fig. 4) [47, 48, 123, 126]. Inflammasome-activated pyroptotic macrophages [181], microbial agents, cytokines and complement factors can further increase TF levels, which can activate the endothelial-glycocalyx and aggravate coagulopathy [177–179]. Wu and colleagues further showed in their mouse sepsis model that inhibition of TF abolishes inflammasome-mediated blood clotting and protects against death [172, 181].

Diagnosing sepsis-induced coagulopathy, like trauma-induced coagulopathy (TIC), has undergone major developments in the last 10 years as whole blood viscoelastic methods have replaced the older unreliable plasmatic methods of prothrombin time (PT), activated partial thromboplastin time (aPTT) and international normalized ratio (INR) [14, 46–48]. *Sepsis-induced coagulopathy is not a static state but a dynamic one with multiple phenotypes that can change over time* (Fig. 4) [14, 47, 48, 100]. In the rotational thromboelastometry (ROTEM) study of Davies and colleagues, they examined 100 ICU patients (50 with sepsis, 20 with severe sepsis and 30 with septic shock) and found increased sepsis severity was associated with shift from a hypercoagulable to hypocoagulable state, with no change in maximum clot firmness [35]. In septic shock, fibrinolysis was markedly impaired towards a bleeding phase, and was significantly associated with 28-day mortality [35]. Anatomopathologic fibrin deposition was not evaluated at autopsy to assess DIC. In 2023, Bui-Thi and colleagues undertook another prospective, observational, single-center study and reported 73% of 161 patients had sepsis/septic shock [14]. ROTEM showed 26% were hypercoagulable, 55% were hypocoagulable, 14% had mixed hypo-hypercoagulation patterns, and 19% were hyperfibrinolytic [14].

The different coagulopathies appear to reflect different timings and severity of infection and sepsis. An early common phenotype in septic patients appears to be a hypercoagulable subtype characterized by prolonged initial clot time with increased maximum clot firmness (MCF) and high fibrinogen levels (Fig. 4). Bui-Thi and colleagues further showed that progression from sepsis to severe sepsis was accompanied by a shift from hyper- to hypo-coagulability with fibrinolysis [14]. The thrombelastography (TEG) study of Luo and colleagues confirmed a hypocoagulopathy in severe sepsis patients which, if present at hospital admission, was an independent risk factor for 30-day mortality [107]. Possible mechanisms responsible for the switch from a hyper- to hypo-coagulopathy are summarized in Fig. 4 [47, 48].

A common mistake in the literature is to equate hypocoagulopathy and fibrinolysis (a bleeding phenotype) with disseminated intravascular coagulopathy (DIC) [47, 48, 100, 125]. DIC is a rare and specific phenotype with diffuse anatomopathologic fibrin deposition in small and mid-size vessels (Fig. 4) [46, 74, 99, 163]. Clinically, DIC continues to be diagnosed using a scoring system involving PT, platelet count, fibrinogen, and D-dimer levels [13, 167, 172]. However, without evidence of intravascular fibrin deposition, diagnosis may not be DIC and impact on optimal patient treatment. We propose the concept of DIC should be confined to a phenotype *with confirmed microvascular fibrin deposits*.

Understanding coagulopathy during sepsis is in its infancy. More research in clinically-relevant animal models and high quality prospective randomized human trials are urgently required to characterize the different early and late phenotypes, and their progression to sepsis and septic shock, as well as sex differences. With more high-quality studies, there is a strong potential that the information can lead to more effective personalized and goal-directed treatments.

### Multiple organ dysfunction syndrome (MODS): not a single event but a systems failure

Of those who die, most are from multiple organ failure which is a still poorly understood consequence rather than the immediate effect of infection.

Coccolini and colleagues (2023) [27]

To highlight the lethality of MODS during sepsis, the 2023 guidelines redefined sepsis as “life-threatening organ dysfunction resulting from a dysregulated host response to infection” [143]. In 1975, Arthur Bauer introduced the term MODS as multiple physiological derangements [8]. Today, MODS is considered a clinical syndrome characterized by the development of progressive and potentially reversible physiologic dysfunction in two or more organs or organ systems [19, 95]. Organ dysfunction syndromes include encephalopathy, acute respiratory distress, myocardial infarction, hepato-renal syndrome, acute necrotizing pancreatitis, acute adrenal insufficiency, rhabdomyolysis, and muscle wasting syndrome (catabolic response) [19, 95].

At the cellular level, MODS develops from persistent tissue hypoperfusion and loss of mitochondrial integrity [49, 54]. ATP is no longer fully replenished leading to ischemia, hypoxia, organ dysfunction, and possible failure. Mitochondrial dysfunction includes decreased proton pumping across the inner mitochondrial membrane, collapsed membrane potential, opening of the mitochondrial permeability transition pore, Ca^2+^ loading, loss of cytochrome C, release of apoptotic-cascade inducing factors, and increased DAMPs and ROS, which exacerbates immune dysfunction, inflammation and coagulopathy [49, 54, 73, 79]. Sepsis also impairs mitochondrial biogenesis and mitophagy, resulting in insufficient renewal of mitochondria, which further impacts cellular respiratory capacity and organ dysfunction. Fever, a manifestation of sepsis, is a result of uncoupling of muscle mitochondria which leads to generation of heat, not energy, and helps to explain muscle wasting despite a high caloric intake [79]. Targeting mitochondrial dysfunction in muscle, and other organs, may offer novel and valuable targets for sepsis.

Mitochondria are not only the cell’s powerhouses. In immune cells, mitochondrial activity regulates their activation, differentiation and survival [94, 102, 103, 114]. Expression of key mediators involved in regulation of mitochondrial function (Sirt1/3, Ampk, Pgc1, Nrf1, Tfam, Mtco3, Nr3c1), for example, are significantly reduced in leukocytes from septic patients [20, 102, 103]. Sepsis-induced mitochondrial dysfunction leads to metabolic reprogramming and altered functional capacity of immune cells, heightened inflammation and immunosuppression [94, 102, 103]. Therapies that target recovery of mitochondrial function may offer a novel approach to reverse leukocyte dysfunction in sepsis [3, 114].

### Cardiovascular dysfunction after sepsis: the puzzle of myocardial depression

There has been a tendency to equate shock, regardless of its origin, with a low cardiac output (CO) and high total peripheral resistance (SVR). While our experience suggests that this is true of hypovolemic and cardiac shock, the same cannot be said of the septic form.

Wilson and colleagues (1965) [180]

Cardiovascular collapse is a major reason for mortality in septic shock patients (Fig. 3) [77]. In the early 1960s, two distinct hypotensive phases of septic shock were characterized; the first was a warm dry skin, tachycardic condition (“warm” shock), and the second phase was a cold clammy skin with a thready pulse with hypotension (“cold” shock) [109]. Using this classification, septic shock initially went through an early hyperdynamic phase (high cardiac output, CO) and either recovered or deteriorated into cardiovascular collapse (low CO) [137]. In 1965, Wilson and colleagues challenged this view by reporting that septic shock patients had normal or elevated CO with low systemic vascular resistance (SVR), and very rarely had a low CO [180]. This was highly controversial and contrary to hemorrhagic/cardiogenic shock, which is characterized by a low CO and high SVR [137]. Using nuclear imaging techniques and thermodilution methods*,* Joseph Parrillo’s group confirmed Wilson’s findings and showed that septic shock patients maintained high CO and low SVR [28, 60, 130]. The group also reported that 75% of patients had a depressed left ventricular ejection fraction (LVEF) in the first few days after the onset of septic shock [130]. Sepsis-induced myocardial depression appears to have all the hallmarks of myocardial stunning after coronary artery bypass surgery [43, 175].

Today, sepsis-induced cardiomyopathy is an acute syndrome of myocardial depression that occurs early after the onset of septic shock and normally resolves in 7–10 days [77]. It occurs in ~ 50% of septic patients and characterized by LV dilatation and depressed LVEF with maintained coronary blood flow [77, 184]. Myocardial depression in sepsis patients is also associated with higher aortic conduit stiffness [64, 87], which may be responsible for reduced venticulo-arterial (VA) coupling and reduced blood flow and O2 to the tissues [135]. VA coupling is defined as the ratio of arterial elastance (Ea) to left-ventricular elastance (Ees), and can be derived from routine echocardiography [67]. The advantage of VA coupling over LVEF or CO is that it provides arterial load properties in addition to LV function [24, 67]. New therapeutics are required to target VA coupling in septic patients, which may prevent myocardial depression and impairment of vascular reactivity [151].

There are at least five main hypotheses of myocardial depression (Table 1). While all five are plausible, they are not mutually exclusive and appear to involve alterations in Ca^2+^ handling, ATP replenishment and myofilament function [9, 17, 64, 70, 77, 93, 115, 131, 138, 145, 159, 161, 182]. The future challenge is finding which one or more of these mechanisms contribute to the decline in myocardial depression and VA uncoupling seen clinically.

**Table 1:** Five hypotheses for sepsis-induced myocardial depression in the setting of inflammation and immune dysfunction. All five are not mutually exclusive

	Hypothesis	Mechanism(s)	References
1	Circulating myocardial depressant(s)	Serum from septic shock patients depressed myocyte contractility in vitro*.* Candidates include bacterial toxins, TNF-α, IL-1β, and interleukin-1 receptor-like 1 (sST2), which may decrease myofilaments’ sensitivity to Ca^2+^ via the induction of excess NO synthesis (blocked by L-NAME). Possible sources of TNF-α and IL-1β are activated monocytes and macrophages.	[120–122, 54, 123–125, 105]
2	Overexpression of cardiac mitochondrial NOS	Excess NO synthesis (and reactive oxygen species), which may partially open the mitochondrial pore, depolarize the inner membrane, and reduce ATP production for contraction.	[126, 114]
3	Myofilament Ca^2+^ responsiveness	Decrease cardio-myofilament Ca^2+^ sensitivity, reduces cross-bridge cycling responsiveness to reduce contractile activation and force development.	[127, 128]
4	Cardiac β-adrenergic desensitization	Cardiac response to sympathetic hyperactivation and ↑catecholamines. Receptor switching from G_s_ to G_i_, which signals β-2 adrenergic receptors to produce a negative inotropic response, presumably by ↓Ca^2+^ availability.	[129]
5	Downregulation of master genes encoding for sarcomeric and mitochondrial proteins	Reduce cross-bridge cycling and ATP availability generated by oxidative phosphorylation.	[105]

*TNF-α* tumor necrosis factor-alpha, *IL-1β* interleukin-1beta, *NO* nitric oxide, *L-NAME* N^G^-nitro-L-arginine methyl ester, *ATP* adenosine triphosphate

### Fluid resuscitation: doing more harm than good?

Recently, the safety of intravenous fluids in patients with sepsis has been called into question with both prospective and observational data suggesting improved outcomes with less fluid or no fluid*.*

Byrne and Van Haren (2017) [16]

**Fig. 5:**
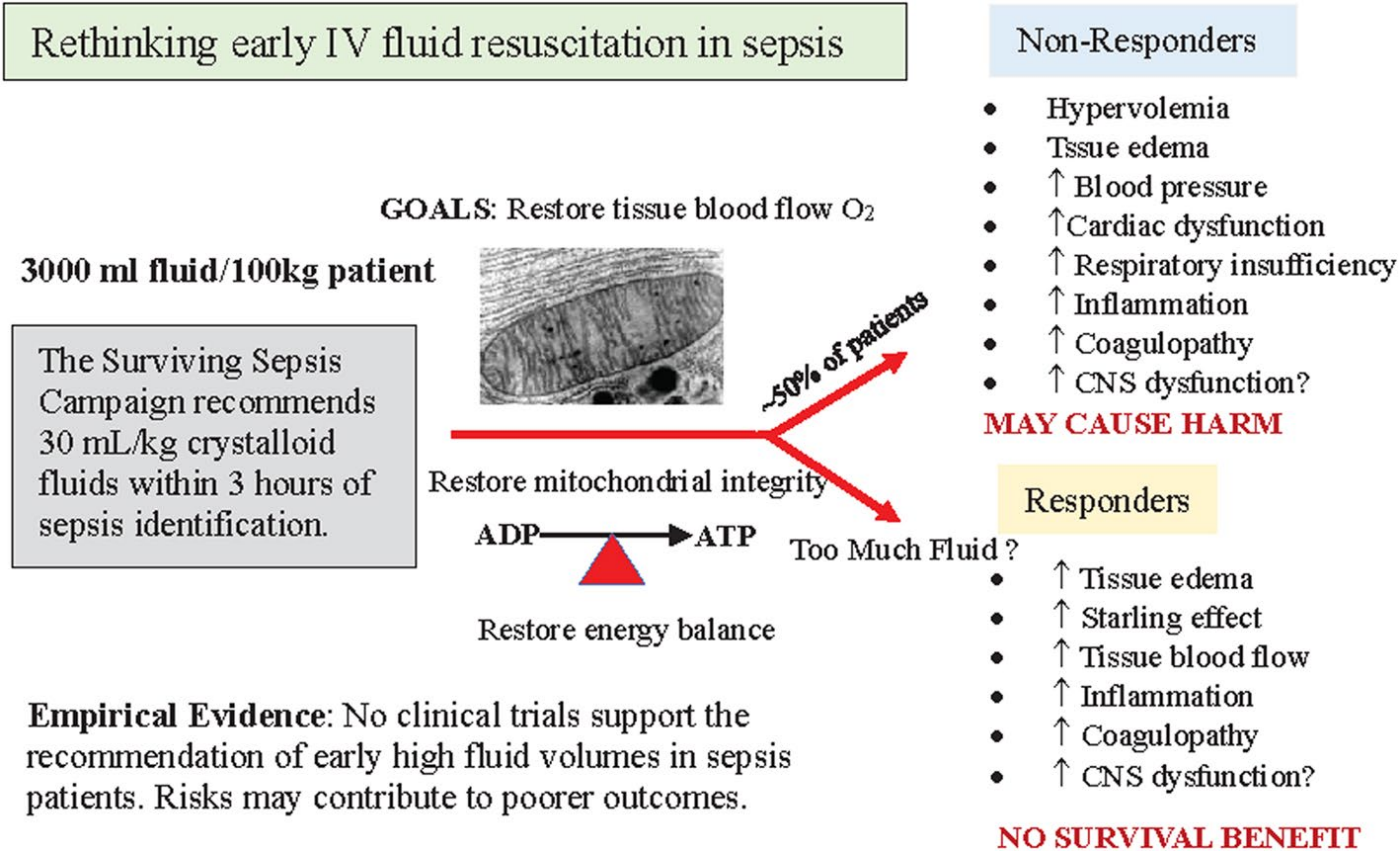
Schematic of 2016–2021 Surviving Sepsis Campaign guidelines that suggest initial resuscitation of at least 30 mL/kg of isotonic crystalloid fluid within the first 3 h of sepsis identification to restore circulating fluid volume and optimize stroke volume. However, there is a paucity of high-quality data to support this clinical practice. The significant heterogeneity of sepsis and the reports that ~ 50% are non-responders makes the recommendation highly problematic. Current evidence indicates that administration of large fluid volumes to the critically ill may cause harm by exacerbating secondary injury (see text). IV, intravenous; CNS, central nervous system; ADP, adenosine triphosphate; ATP, adenosine triphosphate

Current supportive strategies for severe sepsis patients may include an early and goal-directed fluid resuscitation bundle, mechanical ventilation, inotropic and vasopressor therapies, blood cell transfusions, anti-fibrinolytics and mechanical ventilation, and possible renal support [91, 177]. The primary goal of fluid therapy is to reduce dehydration, restore circulating blood volume, optimize cardiovascular function and improve tissue O_2_ (Fig. 5) [16]. Dehydration is common in older adults, often necessitating an initial 500 mL crystalloid bolus [80]. Prior to 2001, use of fluid resuscitation was largely based on historical experience without empirical support from either animal studies or clinical trials [16]. The first supportive evidence for fluid therapy came from the human study of Rivers and colleagues that showed a 16% mortality reduction in septic shock patients [140]. Unfortunately, the survival benefit was not supported in larger independent trials, including the *ProCESS, PROMISE* and *ARISE *trials [108]*.*

Despite the lack of clinical evidence, the 2016–2021 Surviving Sepsis Campaign guidelines continued to promote fluid therapy for septic shock patients (Fig. 5) [58]. The guidelines proposed an initial resuscitation of at least 30 mL/kg of isotonic crystalloid fluid during the first 3 h to restore circulating fluid volume and optimize stroke volume [108]. *Administering an IV fluid volume up to 60% of the normal blood volume to a 100 kg septic patient over 3 h has little or no clinical support and may be associated with higher mortality* [58]. The Surviving Sepsis campaign has now downgraded this recommendation from strong to weak, although the practice appears to continue in many hospitals worldwide [58].

Today, fluid resuscitation may cause harm to some patients. Contrary to its name, normal saline is not normal, and the volumes and timings are not effective in critically ill patients [11, 16, 108, 121, 149, 150]. Large aggressive fluid volumes create dilutional coagulopathy, fluid overload, and pathogenic pulmonary and tissue edema, acute kidney injury, prolonged ICU stays and higher mortality [55, 108, 149, 150]. *If normal saline was evaluated today by the European, USA and Australian regulatory bodies, there is a high chance it was not be approved for human use*. The harmful effects of normal saline solutions were recognized over 100 years ago by George Evans when he wrote in 1911*:* “*One cannot fail to be impressed with the danger of such procedure, if one observes the utter recklessness with which salt solution is frequently prescribed, particularly in the postoperative period, without previous knowledge of the condition of blood pressure, the ability of the heart to handle large amounts of fluid successfully, or the functional capacity of the kidneys to excrete the large amount of chloride thus formed on them*” [5, 57]. More recently, vasopressor agents that increase cardiac contractility or vasoconstriction have also come under clinical scrutiny that include increased cardiac workload, arrhythmias and vasoconstriction-related tissue ischemia [149, 150].

Another vexing problem with the ongoing use of fluid therapies is that up to 50% of patients are non-responders (Fig. 5), meaning they fail to increase preload and stroke volume with IV fluid infusions, and therefore fails to improve tissue O_2_ supply [72, 111]. Unfortunately, there have been few high-quality clinical trials comparing non-responders and responders [55]. Some studies recommend the use of a lung ultrasound, echocardiographic assessment, urine output, and other measures, to prevent fluid overload in the ICU in an attempt to improve patient stratification and optimize treaetment [55].

### Trauma of surgery: a forgotten confounder of poor patient outcomes

It should be remembered always that the patient who has been in shock and resuscitated, and then operated upon, is in a precarious state. His nervous system has been disturbed not only by the original trauma, but also by the low nutrient flow of blood, *and by the surgical procedures incidental to operation (our italics)*.

Walter B. Cannon (1923) Quoted from Traumatic Shock [18] p192

Patients with severe abdominal sepsis often require an emergency laparotomy, which is associated with higher mortality and morbidity compared to less invasive procedures (Fig. 2) [12, 59, 69, 90, 136, 154, 162]. Possible reasons for poorer outcomes include the trauma of surgery itself amplifying immune cell activation, increasing inflammation, coagulopathy and MODS [39, 41]. The extra barrage of DAMPs comes from tissue damage from the first incision, organ manipulation, and surgical correction and drain (Fig. 2) [2]. Torp and colleagues have recently discussed how tissue injury from surgery triggers a generalized inflammatory response and the role mitochondrial DAMPs (mDAMPs) play in worsening its pathophysiology [168]. In addition, surgical site infections occur in up to 35% of patients, which further complicates recovery [2]. Another aspect of the trauma of surgery that is rarely discussed is that the anesthetized brain is still “awake” to the circulating DAMPs from the surgery itself [30, 41], which may pass through the leaky BBB and activate CNS dysfunction and lead to altered mental states [4, 56, 91]. The trauma of surgery aggravates an already precarious state, which is magnified further in older patients with multiple comorbidities [41]. This is an area that requires new therapies to reduce the host’s stress response of surgery [31, 38, 41, 61, 71, 133, 164].

### Future consideration for drug development: from symptoms to system

Clinical study of antiinflammation strategies to treat sepsis has been characterized by a predictable cycle of abundant clinical failures punctuated by an intermittent positive result.

Shapiro and colleagues (2023) [149, 150]

Notwithstanding the ongoing challenges in treating sepsis, great strides are being made in personalized care based on blood biomarkers [143]. Individualized treatments have also progressed in the chronic, immunosuppressive stage of sepsis responsible for later-stage morbidity and mortality [143]. Notwithstanding these advances, there remains few safe and effective drugs for the early treatment of sepsis [92, 102, 103, 149, 149, 150, 150]. For example, there are only a handful of drugs to reduce excessive inflammation and immune dysfunction. Non-steroidal anti-inflammatory drugs (NSAIDs), COX-2 inhibitors and TNF-α inhibitors do not appear to be pro-resolving, and may in fact exacerbate the proinflammatory process [129]. A relative new area of drug design is targeting metabolic reprogramming of immune cells responsible for hyperinflammation and immunosuppression [102, 103]. The future challenge is to safely and effectively translate these immunometabolism-altering drugs to improve patient outcomes in the hospital setting.

Why have there been so few advances in pre-clinical drug development for sepsis and translation to humans? Three possible reasons include: 1) the widespread use of specific-pathogen free (SPF) animal models with their altered microbiomes and immature immune systems that do not represent the human condition [45, 47, 48, 51–53], Conventionally bred and housed animals should be used if human translation is the end-game [51–53], 2) the flawed practice of the treat-as-you go mindset and single nodal drug targeting, which ignores the complexity of the system [49–54], and 3) poor clinical trial design that does not represent the heterogeneity of patient responses to sepsis [21, 143]. With respect to single-nodal targets, we will give one example. It is well established that the IL-1 receptor is a key amplifier of inflammation [149, 150], and its inhibition may resolve the cytokine storm. A drug that inhibits the IL-1-receptor, anakinra, has an excellent safety record in humans, however, it has failed to show a survival benefit after sepsis or COVID-19 [149, 150]. The ‘single-step’ drug target approach can be traced back to the molecular revolution of the 20th century, which began in around 1953 after the discovery of DNA [51–53]. Nobel Laureate Sir Francis Crick embodied this highly mechanistic mindset when he wrote “the ultimate aim of the modern movement in biology is to explain all biology in terms of physics and chemistry” [29]. From a molecular standpoint Crick was correct, however, its relevance to the workings of the whole body has not kept pace [51–53]. The advent of the “Omic” technologies to drill deeper into molecular mechanisms has occurred at the expense of systems analysis. *Reductionism is important in breaking a complex system into its simpler parts, but it does not do away with the system*. New systems-based therapies are urgently required to treat sepsis.

What would a systems-based drug look like? Ideally, a systems-based drug would blunt the CNS-linked feedback circuits (or axes) that drive secondary injury and poor patient outcomes. The drug would reduce the CNS stress response, maintain BBB integrity, promote CNS-cardiovascular coupling, prevent myocardial depression, protect the endothelial-glycocalyx, reduce excessive inflammation, reduce immune dysfunction, correct coagulopathy, and deliver sufficient O_2_ to tissue mitochondria [49, 50, 54]. The drug might also involve reprogramming immune cell metabolism and placing a ‘brake’ on the hyperinflammatory response and permitting anti-inflammatory processes to resolve the host’s responses to sepsis. No such systems-acting drug exists.

We have been developing an adenosine, lidocaine and magnesium (ALM) fluid therapy for hemorrhagic shock, traumatic brain injury (TBI) [44, 50–53], burns [34, 51–53], orthopedic trauma [118, 119] and the trauma of surgery [33]. We have shown the drug shifts sympathetic hyperactivity to parasympathetic dominance in the rat model of non-compressible hemorrhagic shock [96], restores cardiac output [97], protects against endothelial glycocalyx shedding with 97% rapid restoration [169], and reduces the incidence of MODS [50, 98]. During resuscitation, we have shown the ALM therapy is neuroprotective at hypotensive pressures (MAPs 47–50 mmHg), which has important implications for sepsis [50–53, 97, 98]. This data has been use to formulate a Systems Hypothesis of Trauma (SHOT), which may be applicable for sepsis and septic shock [49, 50, 54].

Our proof-of-concept studies in a rat polymicrobial sepsis model [65, 66] and pig lipopolysaccharide (LPS)-endotoxin model [63] have been encouraging. In the pig LPS model, ALM therapy induced a profound and reversible hypotensive state (MAP 47 mmHg) by maintaining CO and lowering SVR with little or no change in tissue O_2_ delivery [63]. CO was maintained by preserving preload recruitable stroke work and improving VA coupling [63]. *Importantly, systemic hypotension is not deleterious if CO, VA coupling and O*_*2*_* delivery to the tissues are maintained by lowering SVR* [51–53]*.* This ALM-induced hypotensive state involves re-setting the CNS-control of O_2_ delivery to tissue mitochondria, that may be beneficial to sepsis patients [50]. Similarly, ALM therapy induced a reversible hypotensive state in a rat model of polymicrobial sepsis with reduced pulmonary edema and correction of coagulopathy [65]. ALM led to 88% survival after six days, without antibiotics, whereas saline controls died early from inflammatory/immune dysfunction and multiple organ failure [66]. Importantly, the combination of ALM is key to whole body protection whereas the individual actives, A, L or M are not [50]. While appreciating the success rate of translating new drugs to humans is less than 5% [147], further ALM preclinical sepsis studies are required to facilitate translation to human safety trials.

## Conclusions

The clinical management of sepsis appears to be at a crossroads and requires a new revolution to better understand how the body responds to an infection, new markers to detect its progression and new system-acting drugs to treat it. The current targeting of any single step along a signalling pathway has not been successful because it ignores the complexity of the system. We argue if the immune-driven, CNS-sympathetic hyperactivation can be suppressed, cardiovascular-endothelial glycocalyx coupling will be improved, O_2_ delivery will be maintained, and secondary injury, including hyperinflammation and immunosuppression, will be prevented leading to better clinical outcomes. We are developing a systems-based drug in animal sepsis/endotoxemia models, which may confer whole body protection in humans.

## Data Availability

The datasets during and/or analysed during the current review can be made available from the corresponding author on reasonable request.
